# Culture models to study leukocyte trafficking across the choroid plexus

**DOI:** 10.1186/2045-8118-10-1

**Published:** 2013-01-10

**Authors:** Tobias Tenenbaum, Ulrike Steinmann, Corinna Friedrich, Jürgen Berger, Christian Schwerk, Horst Schroten

**Affiliations:** 1 Paediatric Infectious Diseases, Department of Pediatric and Adolescent Medicine, Universitätsmedizin Mannheim, Medical Faculty Mannheim, Heidelberg University, Theodor-Kutzer-Ufer 1-3, Mannheim, Germany; 2 Institut für Genetik von Herzerkrankungen (IfGH), Department für Kardiologie und Angiologie, Universitätsklinikum Münster, Münster, Germany; 3 Max Planck Institute of Developmental Biology, Tuebingen, Germany

**Keywords:** Blood-cerebrospinal fluid barrier, Leukocyte, Transmigration, Meningitis

## Abstract

**Background:**

A critical point during the course of central nervous system infection is the influx of leukocytes from the blood into the brain across the blood-brain barrier (BBB) and the blood-cerebrospinal fluid barrier (BCSFB). However, experimental *in vitro* models to investigate leukocyte transmigration across cultured choroid plexus epithelial cells have been lacking so far.

**Methods:**

We have developed a porcine and human “inverted” culture insert system that enables leukocyte transmigration specifically from the physiologically relevant basolateral side. The models use primary porcine choroid plexus epithelial cells (PCPEC) and human choroid plexus papilloma cells (HIBCPP). As a prerequisite for a functional barrier, we optimized culture conditions in which cells are maintained in serum-containing medium until high barrier function is reached. Leukocyte transmigration through the plexus epithelial cells is analysed by three-dimensional Apotome^®^-imaging and electron microscopy, and the route of transmigration through the plexus epithelial cells, i.e. transcellular as well as paracellular, can be determined.

**Discussion:**

As a functionally relevant porcine and human BCSFB model, PCPEC and HIBCPP respectively, offer a wide range of options for analysis of disease-related mechanisms at the choroid plexus epithelium, especially involving human pathogens. Moreover, our *in vitro* models facilitate the investigation of leukocyte entry into the CNS via the blood-CSF barrier.

## Background

The central nervous system (CNS) is separated from the blood by specific cellular structures including the blood-brain barrier (BBB) and the blood-cerebrospinal fluid (CSF) barrier (BCSFB). Whereas the endothelial cells of the brain microvasculature form the BBB, the structural basis for the blood-CSF barrier is the choroid plexus epithelium. The epithelial cells of the choroid plexus are closely connected to each other by a belt of tight junctions (TJs). TJs of epithelial and endothelial cells maintain the asymmetry of the plasma membrane and serve as a regulated permeability barrier for paracellular transepithelial and transendothelial transport of physiologically important solutes, the penetration of microorganisms and other antigenic material [1]. Other important functions of the choroid plexus are maintenence of homeostasis in the CNS and CSF-secretion and participation in neurohumoral brain modulation and neuroimmune interactions [2,3]. In the case of the endothelium at the BBB**,** these cells are interconnected by a dense network of TJs and they exhibit a low pinocytotic activity concomitant with the absence of fenestrae [4]. Properties of these cellular barriers are a high transendothelial or transepithelial electrical resistance (TEER) as well as a low permeability for macromolecules [5].

There is increasing evidence that the choroid plexus plays an important role in CNS inflammation, but the exact role of the BCSFB in this context is still under investigation [6,7]. The most comprehensively-investigated model for CNS inflammation is experimental autoimmune encephalomyelitis (EAE), which is considered the prototype model for the human disease multiple sclerosis (MS) [3]. But also bacterial and viral meningitis are important causes of mortality and morbidity despite advances in antimicrobial therapy. For many important meningitis pathogens such as *Neisseria meningitides* (*N. meningitides*) [8], *Haemophilis influenzae* (*H. influenzae*) [9], *Escherichia coli* (*E. coli*) [10,11], *Listeria monocytogenes* (*L. monocytogenes*) [11], *Streptococcus suis* (*S. suis*) [12] and enteroviruses [13], experimental data suggest involvement of the choroid plexus during bacterial entry into the brain. Most cases of meningitis develop as a result of haematogenous spread, but it is unclear how circulating bacteria cross the blood-brain barriers. Moreover, the mechanisms by which pathogens enter the CNS, lead to inflammation, pleocytosis (with predominantly polymorphonuclear neutrophils (PMN) in bacterial and lymphocytes/monocytes in viral meningitis) blood-brain barrier disruption and neuronal injury are still under investigation [14,15].

During inflammatory events the BBB and BCSFB undergo major alterations, which lead to an opening of TJs, break-down of barrier function and massive influx of immune system cells into the brain [16]. An important factor for investigating this disease is the development of suitable *in vitro* systems mimicking the above-mentioned barriers. Whereas human models of the BBB employing immortalized cell lines have been developed [17-19], *in vitro* systems mimicking the BCSFB are limited to animal models, including rat cell lines and primary porcine choroid plexus epithelial cells (PCPEC) [5,20-23] (an extensive recent review covering BCSFB *in vitro* models is provided by Strazielle and Ghersi-Egea [24]).

The cultivation of PCPEC in medium with reduced serum concentrations improves the morphological polarity of plexus epithelial cells as well as the barrier forming cell-cell interactions. This can be clearly demonstrated by an increase in transepithelial resistance (TEER) values and a decrease in macromolecular permeability [5]. We have used this culture system for the first time to study the pathogenesis of bacterial meningitis, and ideally use *S. suis* as a relevant pathogen causing disease in pigs and humans. *S. suis* is a well characterized swine pathogen causing a wide range of infections such as meningitis, septicemia, arthritis and pneumonia [25].

Recently a human choroid plexus papilloma cell line (HIBCPP) was established [26] and has now been characterized for its suitability as BCSFB model system by our group [27]. It was demonstrated that HIBCPP, when cultured under appropriate conditions, displays several features of a functional BCSFB including the formation of TJs and the development of a high TEER concomitant with a low permeability for macromolecules when grown on cell culture inserts.

To study the pathogenesis of CNS inflammation at the porcine and even more relevant the human BCSFB, the development of an inverted culture system was necessary. By this means the investigation of the physiological interaction of pathogens or leukocytes with the basolateral cell side of the choroid plexus epithelial cells is possible. In this study protocol article we describe the experimental setup of our inverted BCSFB models and the possibilities of experimental analysis with this system.

## Methods

**Figure 1:**
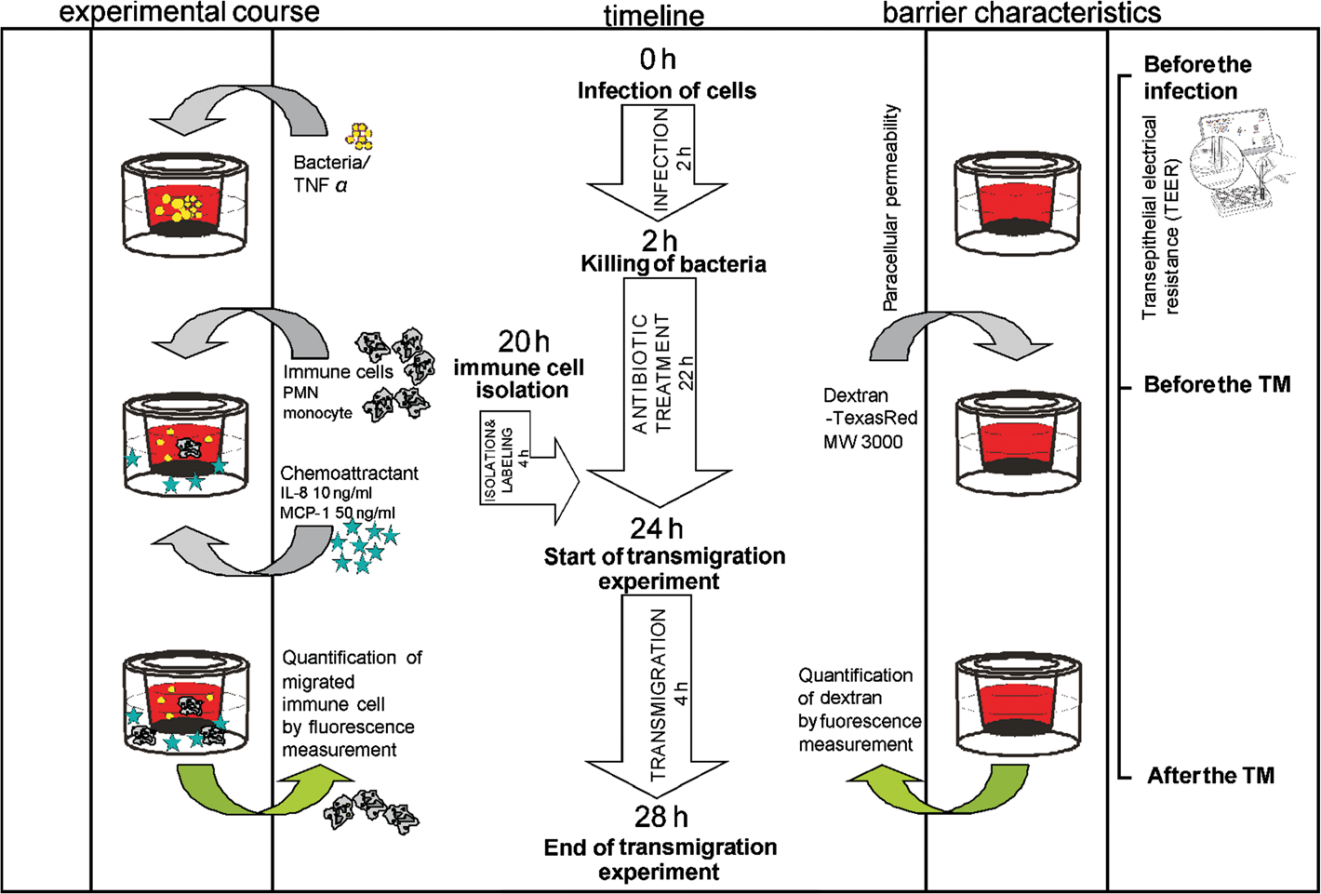
**Experimental setup of the analysis of leukocyte transmigration in porcine and human inverted culture system with PCPEC and HIBCPP, respectively. **The image of the Ohm voltohmmeter is a courtesy of EMD Millipore cooperation. Abreviations: IL-8 = Interleukin-8; MCP-1 = monocyte chemotactic protein-1; MW = molecular weight; TM = transmigration.

In this section we describe our experimental setup for analysis of leukocyte transmigration in BCSFB *in vitro* models as summarized in Figure 1. In brief, after seeding and culturing PCPEC on the basolateral side of the culture insert membrane, cells are stimulated and barrier function and morphology analyzed for the duration of the experiment. Leukocyte transmigration is determined in the presence or absence of a chemoattractant by fluorescence quantification and imaging. Each experimental step is described in the following paragraphs in more detail.

### Preparation and cultivation of PCPEC on inverted cell culture inserts

Epithelial cells from porcine choroid plexus are obtained by a modified preparation protocol [21]. Brains from freshly slaughtered pigs are dissected and the choroid plexus tissue from the lateral and the fourth ventricles removed, transported with HBSS in Ca^2+^/Mg^2+^ (Gibco BRL, Eggstein, Germany) and HEPES buffer (BioWhittaker, Apen) to the laboratory, and treated hereafter with mixed cold and warm trypsinisation (0.2% solution, Biochrom, Berlin, Germany, 45 min. at 4°C, 20 min. at 37°C). After termination of the trypsin digestion with an appropriate volume of fetal calf serum (FCS) the cells are centrifuged at 20 × g and resuspended in DMEM/HAM’s F12 1:1 (Sigma-Aldrich, Deisenhofen, Germany) supplemented with 4 mM L-glutamine (Gibco BRL, Eggstein, Germany), 10% heat-inactivated FCS (Biochrom KG seromed, Berlin, Germany), 5 μg/ml insulin (Sigma-Aldrich, Deisenhofen, Germany), and penicillin (100 U/ml)/streptomycin (100 μg/ml) (ICN Biomedicals GbbH, Eschwege, Germany). The medium is supplemented with 20 μM of cytosine-arabinoside (Cell Pharm GmbH, Hannover, Germany) to suppress the growth of contaminating fibroblast-like cells. The cells are seeded on laminin (BD, Le Pont De Claix, France) coated cell culture inserts (pore diameter 3.0 μm, 0.33 cm^2^; Falcon, BD, Le Pont De Claix, France) that are flipped over and placed in a medium flooded 12-well plate. For laminin-coating, 35 μl of a 50 μg/ml solution of laminin are applied to lower filter side with subsequent drying overnight. Cells are concentrated in 100 μl medium, using a seeding density of 60 cm^2^/g wet weight of choroid plexus tissue, are fed the following day and the culture insert are flipped over again on day 2 after plating. Upon confluence, PCPEC have a seeding density of approximately 1 × 10^5^ cells/cm^2^ (evaluated by DAPI staining of the cell nuclei using immunofluorescence imaging as described below). In the following cells are cultivated until reaching a TEER ~ 110 Ω × cm^2^, hereafter switched to medium with FCS 1%, and finally used for the experiments 3-5 days later when TEER becomes greater than 200-300 Ω × cm^2^.

### Cultivation of HIBCPP on cell culture inserts

HIBCPP are cultured in DMEM/HAM's F12 1:1 supplemented with 4 mM L-Glutamine, 5 μg ml^−1^ insulin, penicillin (100 U/ml) and streptomycin (100 μg/ml), 15% heat inactivated fetal calf serum (FCS) [HIBCPP-medium with 15% FCS]. Since HIBCPP have been described to change doubling time with increasing passages [26] only cells between passage 33 and 37 are used. For culture insert-based assays the cells are seeded on cell culture inserts (pore diameter 3.0 μm, pore density 2.0 × 10^6^ pores per cm^2^, membrane diameter 0.33 cm^2^; Greiner Bio-One, Frickenhausen, Germany). Since HIBCPP can form papillary-like structures and grow in multilayers [26], seeding of HIBCPP needing extensive trypsinization to allow formation of a maximal proportion of a monolayer on the cell culture inserts. Cells are trypsinized with trypsin 0.25% (Live technologies, Darmstadt, Germany) for 12-15 min (up to 25 minutes) at 37°C, washed hereafter and seeded onto filters at a seeding density of 4 × 10^4^/well or 2 × 10^5^/ well depending on the time point planned for the experiment . Subsequently, cells are washed once each of the following two days. Medium is not added to the lower well before day two after seeding. For the inverted cell culture insert system the cells are basically treated as described above for PCPEC [7]. Upon confluence, HIBCPP have a seeding density of approximately 1.21 × 10^6^ cells/cm^2^ (evaluated by 4,6-diamidino-2-phenylindole staining of the cell nuclei using immunofluorescence imaging). When TEER values become greater than 70 Ω × cm^2^, cell culture is continued in HIBCPP-medium containing 15%, 1% or 0% FCS. Cells can be used for experiments 1 or 2 days later when the TEER is around 500 Ω × cm^2^.

### Measurement of transepithelial electrical resistance (TEER)

Barrier properties of PCPEC monolayers are documented by measuring TEER. TEER can be determined using an epithelial tissue voltohmmeter (EVOM^®^, World Precision Instruments, Sarasota, FL, USA) and an STX-2 electrode system. When TEER values reach more than 200-300 Ω × cm^2^, PCPEC inverted cultures are suitable for use. In PMN transmigration experiments, TEER is monitored for 4 h. The electrical resistance for cells in medium alone is used as the negative control and remains stable during all experiments.

### Determination of paracellular permeability

Paracellular permeability across cell monolayers in the basolateral-to-apical direction is determined during PMN transmigration experiments using Texas Red-labelled dextran (MW 3000; Sigma, Deisenhofen, Germany). For this purpose, Texas Red-dextran (TR-dextran, 100 μg/ml) is loaded into the upper compartment prior to the incubation period. At different time intervals, samples from the lower compartment can be collected and fluorescence measured in a Tecan Infinite M200 Multiwell reader (Tecan, Switzerland). The percentage of dextran flux is calculated in relation to an internal standard. TEER and permeability measurements can be performed with the same cultures as used for PMN transmigration.

### Isolation of PMN

For the PMN transmigration assay, blood is taken from freshly slaughtered pigs at the abattoir following the ethical guidelines. The blood is collected into sodium**-**heparin coated tubes (2500U/50 ml blood). Hereafter, PMN are isolated from non-coagulated citrate blood by Percoll density sedimentation according to the manufacturer’s instructions (Biochrom, Berlin, Germany). Contaminating erythrocytes are lysed with NH_4_Cl on ice. After centrifugation (10 min, 300 g, 4°C), the lysed erythrocytes are removed and the PMN pellet is washed with PBS. PMN are resuspended in culture medium at a cell density of 1 × 10^7^/ml. For transmigration assays, PMN are loaded with the fluorochrome 2’,7’-bis-(2-carboxyethyl)-5-(and-6)-carboxyfluorescein, acetomethyl ester (BCECF-AM; Molecular Probes, Eugene, OR, USA) at a concentration of 1 μM for 15-30 min at 37°C according to the manufacturer’s instructions. After the staining period, cells are washed with culture medium and counted with a möLab cell counter (möLab, Langenfeld, Germany).

### Stimulation of PCPEC

In our experimental setup, PCPEC are stimulated with either TNFα (R&D Systems, USA) from the apical and basolateral side (10 ng/ml) for 24 h or basolaterally (blood-side) infected with *S. suis* strain 10 with an multiplicity of infection (MOI) of 10 and hereafter incubated for 2 h at 37°C and 5% CO_2_. After the incubation period penicillin/streptomycin (100 U/ml/ 100 μg/ml) is added to the upper and lower compartment of the cell culture insert to inhibit further extracellular bacterial growth and therefore to prevent cytotoxic effects. PMN transmigration assays are performed after an additional 22 h on the following day.

### PMN transepithelial migration assay

For transepithelial migration assays, BCECF-AM-loaded PMN are added to the upper cell culture insert compartment (blood-side) of control, TNFα or *S. suis* stimulated cells in a PMN:PCPEC ratio of 10:1. As chemoattractant IL-8 (R&D Systems, USA) is used at 10 ng/ml and added to the lower cell culture insert compartment (CSF-side) 30 min before starting the transmigration experiments. After 4 h of transmigration the cell culture inserts are removed and the 24-well plates are centrifuged (5 min, 300 x g) to ensure that all PMN are attached to the bottom of the wells. The supernatants are collected for permeability measurements. The PMN are washed once with HBSS with Ca^2+^/Mg^2+^ and again centrifuged (5 min, 300 x g). Transmigrated PMN are lysed by 1% Triton X-100 in PBS and quantified by fluorescence measurement with a Tecan 200 M Infinite Multiwell reader (Tecan, Switzerland) in relation to an internal standard.

### Immunofluorescence

Confluent PCPEC are grown on inverted cell culture inserts, stimulated with *S. suis* or TNFα and co-cultured with PMN as described above. After 4 h of transmigration towards a gradient of IL-8, the cells are washed, fixed with formaldehyde 4% (10 min) and hereafter washed again. The filters can principally be stored at 4°C until final staining. Filter membranes are cut out of the filters and cells are permeabilized with 0.5% Triton X-100/1% BSA in PBS at room temperature for 1 h. Subsequently, the filters are washed again with PBS without Ca and Mg and incubated overnight at 4°C with the primary antibodies to stain the TJ proteins. Occludin or ZO-1 rabbit antibodies (1 μg/ml) are used at a dilution of 1:250 (Zymed Laboratories, San Francisco, USA). On the following day the cells are washed again, incubated for 60 min with the secondary antibody 1:1000 (Alexa fluor^®^ 594 goat anti-chicken; Molecular probes, Karlsruhe, Germany), with Phalloidin Alexa fluor^®^ 660 (Invitrogen, Paisley, UK) for staining the actin cytoskeleton and with 4‘-6-diamidino-2-phenylindole dihydrochloride (DAPI) (Calbiochem, Merck KGaA, Darmstadt, Germany) (1:25.000) for staining nuclei. PMN are labelled with the granulocyte-monocyte marker SWC3a-FITC (1:100 in PBS) (Southern Biotech, Birmingham, AL, USA) for 30 min. After washing the cells three times with PBS the culture inserts are embedded in ProLongAntifadeReagent (Invitrogen, Karlsruhe, Germany). Images are acquired with Zeiss Apotome^®^ and Axiovision software (Carl Zeiss, Jena, Germany) using a 63x/1.4 NA objective lens. The image acquisition is carried out using the Zeiss scanning software Axiovison 4.6 and Axiovison module Inside 4D.

### Scanning electron microscopy

Samples are fixed with 2.5% glutaraldehyde in cacodylate buffer, postfixed with 1% osmium tetroxide in phosphate-buffered saline, dehydrated in a graded series of ethanol and critical-point-dried with CO_2_. Finally the samples are sputter-coated with a layer of 7 nm gold/palladium (Bal-Tec MED 010) and examined at 20 kV accelerating voltage in a Hitachi S-800 field emission scanning electron microscope.

### Troubleshooting

Several factors have to be taken into consideration, if implausible results in the transmigration experiments with PCPEC or HIBCPP are observed. Preferentially, freshly-isolated PMN should be used to guarantee a high cell viablility**.** In case of a high red blood cell contamination, a second step of erythrocyte lysis should also be performed. However, the lysis should be not perfomed for too long, since it may otherwise also endanger cell viablility. A TEER between 200-300 Ω x cm^2^ in the case PCPEC and a TEER between 200-500 Ω x cm^2^ in the case of HIBCPP are optimal for performing the transmigration experiments. Lower or high values may deliver sub-optimal results. Daily monitoring of TEER guarantees determination of the time-point most suited for the start of experiment. Before a bacterial infection experiment is initiated, the filters have to be thoroughly washed with antibiotic-free medium.

## Discussion

One crucial step in the pathogenesis of inflammatory diseases of the CNS is the excessive infiltration of leukocytes into the CSF bearing severe consequences. To study the interaction of pathogens or leukocytes with the basolateral cell side of the choroid plexus epithelial cells *in vitro*, the development of an inverted cell culture system became necessary.

Only a few inverted culture insert systems exist so far that enable investigation of leukocyte transmigration across cellular barriers. Most of the models use epithelial cell lines although primary cells are occasionally used also [28]. In general, leukocytes isolated from peripheral blood can be added to the upper chamber of the culture insert and stimulated to migrate toward a chemoattractant [e.g., n-formyl-methionyl-leucyl-pheny- lalanine (fMLP), interleukin (IL)-8, C5a, leukotriene B4, IL1β, MCP-1, CXCL-12] applied to the lower reservoir. Depending on which side of the filter the cells are cultured, transmigration can be modelled in either the apical-to-basolateral or the basolateral-to-apical direction. The surface area and filter pore sizes can be varied, but typically filters with a surface area of 0.33 cm^2^ and pore sizes of 3.0–5.0 μm-large enough to allow the passage of leukocytes-are used. Culture inserts with pore sizes that are too small to allow leukocyte transepithelial migration to occur may be used to determine the effects of soluble factors or intercellular contact on cellular functions [29-32].

Recently, we established an inverted cell culture insert model of PCPEC and HIBCPP that display robust barrier properties and enable investigation of pathogen and leukocyte transmigration in the physiologically- relevant basolateral-to-apical direction [7,27]. Of note, we also established a novel porcine choroid plexus epithelial cell line (PCP-R), which displays important features of a functional BCSFB *in vitro* model, i.e. the formation of a strong barrier function as demonstrated by a high TEER and a low permeability for macromolecules [33]. In preliminary experiments, this cell line seems also to be suitable for the establishment of an inverted cell culture system.

As a model of an inflammatory event in the CNS, we established bacterial infection with the zoonotic pathogen *S. suis* mimicking bacterial meningitis in our porcine model. Here, we could demonstrate that these bacteria specifically invade PCPEC from the basolateral side in a capsule-dependent manner [7]. Inflammatory activation of epithelial and endothelial cells, e.g. after bacterial infection, induces the release of interleukin-8 (IL-8/CXCL8) and other chemokines that recruit polymorphonuclear neutrophils (PMN) [28,34]. The chemokine IL-8 is known to interact with its cognate receptors CXCR1 and CXCR2. CXCR2 is the main receptor involved in neutrophil chemotaxis, leading to cell migration into the brain during injury, infection or disease [35]. Therefore IL-8 seemed to be a suitable chemoattractant to study PMN transmigration at the BCSFB in our model *in vitro*. In fact, IL-8 induced a strong chemotaxis in our transmigration experiments [36].

**Figure 2:**
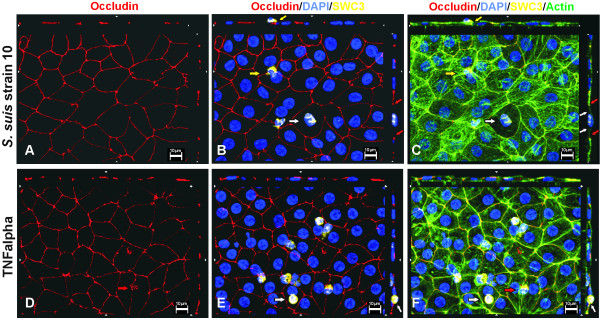
**Transmigration of PMN across PCPEC after stimulation with *S. suis *or TNFα. **En face Apotome microscopy of monolayers stained for occludin (red), actin (phalloidin, green) and nuclei (DAPI, blue) after PMN (SWC3a-FITC, yellow) transmigration. Top and side of each panel is a cross section through the z-plane of multiple optical slices. **A-C.***S. suis *stimulated PCPEC. PMN is localized within the cell body, surrounded by tight junctions (red arrows) and actin (white arrows). Another PMN is leaving the cell close to the tight junction (yellow arrows). Moreover tight junction and actin cytoskeleton disarrangement is demonstrated. **D-F. **TNFα-stimulated PCPEC. Several PMN close to tight junctions and in the middle of PCPEC cell body are demonstrated. One PMN is about to leave the cell via the apical membrane (white arrows). Another PMN seems to transmigrate via the paracellular route, since the occluding morphology is strongly altered in the area the PMN is transmigration through (red arrows). This figure is a representative example of three independent experiments that all gave similar results. Scale bar, 10 μm.

**Figure 3:**
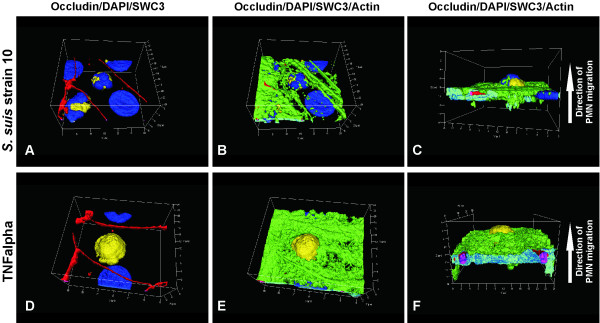
**3**-**D Analysis of PMN transmigration across PCPEC. **Three-dimensional immunofluorescence images of stimulated PCPEC co-cultured with PMN were reconstructed from 0.3 μm Apotome optical sections, using Zeiss software Inside 4D. **A-C.***S. suis *stimulated PCPEC. Views from above on the apical cell side (**A**,**B**) show transcellular PMN migration, in which the centrally localized PMN migrates distal to the TJ. The sideview indicated that the PMN is about to leave the cell (**C**). **D-E. **TNFα-stimulated PCPEC. A centrally localized PMN (**A**,**B**), that transmigrates from the basolateral to the apical direction (**C**) is demonstrated. This figure is a representative example of three independent experiments that all gave similar results. Scale bar, as indicated.

**Figure 4:**
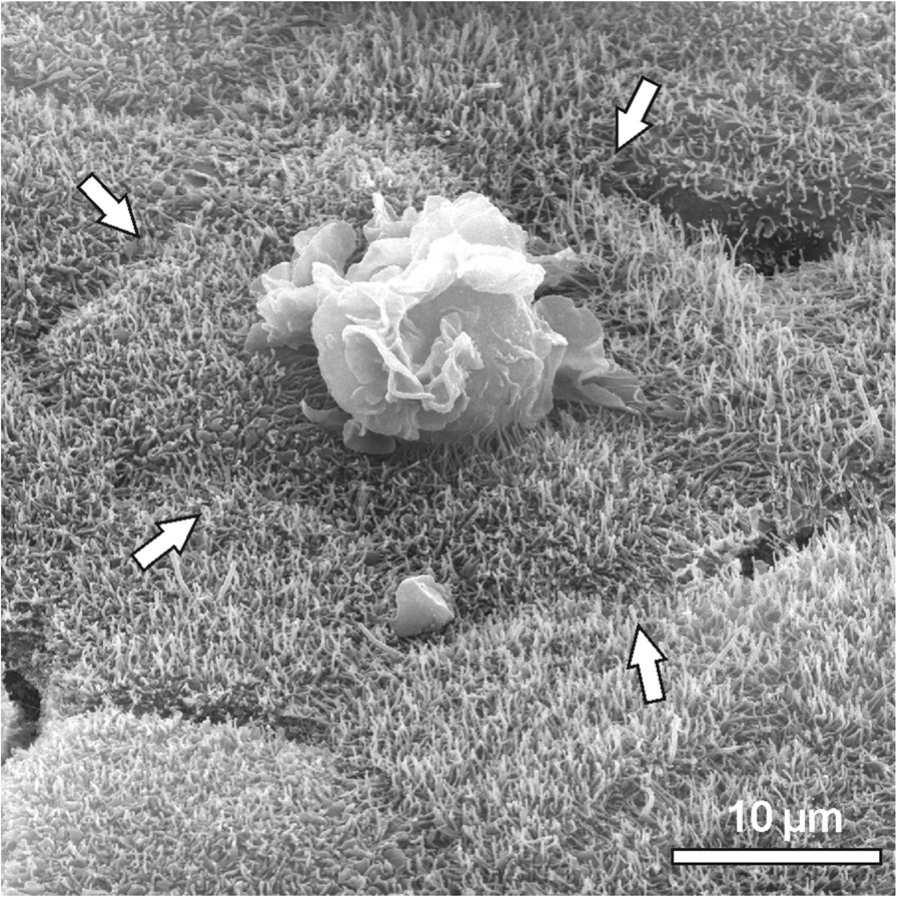
**Scanning electron microscopical view of PMN travelling through the PCPEC monolayer.** PCPEC were stimulated with *S. suis *strain 10. The scanning electron microscopical view shows the transmigrating PMN is clearly distant to the intercellular borders (white arrows).

Employing immunofluorescence and electron microscopic studies we could now show for the first time in an *in vitro* model of the BCSFB that PMN can traverse epithelial monolayers transcellularly. An example for analysis by immunofluorescence is shown in Figure 2, demonstrating that PMN are either localized within the cell body of stimulated PCPEC, surrounded by tight junctions and actin, or are found close to tight junctions. Rarely, tight junction disarrangement is demonstrated in areas of PMN transversal. Views from above on the apical cell side in 3D images show in detail transcellular PMN migration, in which the centrally localized PMN migrates distal to the TJ. The sideview indicates that the PMN is about to leave the cell (Figure 3). Extensive sequential analyses of the PMN transmigration process with Apotome^®^-imaging and electron microscopy revealed that paracellular migrating PMN stop just before tight junctions. Interestingly, PMN subsequently appeared to proceed by transcellular migration via funnel-like structures developing from the apical membrane [36]. Moreover, in scanning electron microscopic analysis we frequently observed PMN in clear distance to intercellular borders (Figure 4). Unlike for transendothelial migration, there was until recently no evidence that PMN take the transcellular route through epithelial cells. For transendothelial migration the paracellular route between adjacent cells was postulated for a long time, but in the meanwhile the transcellular route directly through the endothelial cell body has been well documented [37,38]. The exact mechanisms for the transcellular migration of PMN through PCPEC, especially the roles of adhesion and tight junction molecules, remain to be elucidated further.

Of note, in the human BCSFB model, a polar invasion of *N. meningitidis* from the physiologically-relevant basolateral cell side could also be demonstrated [27]. Currently, experiments are in progess in the human inverted cell culture system to analyze the mechanisms of PMN and monocyte transmigration across HIBCPP after infection with *N. meningitidis*. In the same model, we recently analysed the permissiveness of plexus epithelial cells for *Echovirus 30* (EV30). Analysis of the cytokine/chemokine-profile following enteroviral infection with a cytometric bead array and Q-PCR revealed an enhanced secretion of PanGRO (CXCL1, CXCL2 and CXCL3), IL8 and CCL5. However, there was only a minor effect of HIBCPP-infection with EV30 on transepithelial T lymphocyte migration with or without the chemoattractant CXCL12. Moreover, CXCL3 did not significantly enhance T cell migrations [39].

In the following we describe and discuss the differences between the known inverted culture transwell models that study leukocyte transmigration *in vitro.* Epithelial monolayers such as intestinal epithelial T84 cells have been shown to grow in upright or inverted fashion on collagen-coated permeable supports. In order to grow the T84 on the filter bottom the culture insert cup was inverted and fitted with a tight plastic collar then placed in a bath of medium, which was added also into the collar. T84 cells were harvested and cells were added to the collagen-coated surface of each culture insert which was then left to further incubate. The collars were subsequently removed and the culture inserts righted and placed into a 24-well plate with fresh media [29-31]. Since plastic collars seem easy to handle this tool has frequently been used.

In a lung *in vitro* model, human airway mucoepidermal cell line H292 or human primary bronchial epithelial cells (HBEC) were also cultured as inverted monolayers [40], according to Parkos and colleagues [29], with minor modifications [41]. Apical stimulation of the monolayer was performed with IL-lß and PMN were labelled with ^51^Cr. The fluids from both chambers as well as that of the culture inserts were collected separately after the transmigration experiment and hereafter the amount of ^51^Cr was determined in a gamma counter, which has the advantage of precise analysis, but also disadvantage of the requirement for radioactive facilities [40]. Calu-3 cells as well as primary human ATII cells were also previously used in inverted culture insert systems [34]. Here, the chemoattractant fMLP was added to the lower chamber. After migration, epithelial cells were purified from PMN by magnetic-activated cell sorting separation using anti–epithelial cell adhesion molecule antibodies. This form of analysis requires special equipment and may not be applicable for broad use. Another lung infection model consisting of alveolar epithelial cells (A549) and human PMN grown on inverted culture inserts was used to determine whether also bacteria such as *Pseudomonas aeroginosa, Klebsiella pneumonia* and *E. coli* are capable of inducing PMN migration across these epithelial barriers [42]. PMN that fully migrated into the apical chamber were also quantified by the myeloperoxidase assay as used by others [32,43].

To optimize the lung *in vitro* model, Mul *et al*. developed a bilayer transmigration model composed of primary human endothelial (human papilloma virus-immortalized HUVEC cell line or freshly isolated, primary HUVECs) and lung epithelial cells (H292 or primary bronchial epithelial cells), simultaneously cultured on opposite sides of culture inserts [44]. PMN were labelled with calcein-AM prior to the start of the transmigration assay and after lysis the amount of fluorescence in each of culture insert compartments or attached cells was measured in a spectrofluorimeter at the end of the experiment. All in all, bilayer models are more *in vivo* like, than single cell models, and therefore may be preferred. However, from our experience, establishment of such a system is demanding, since culture conditions may differ significantly among the cells and may lead to interference.

In contrast to the described inverted systems above PCPEC were cultured on laminin coated culture inserts and no plastic collar was used. PMN were labelled with BCECF-AM that can easily determined by fluorescence analysis. Since measurement of fluorescence is easy to perform, labelling of PMN with a fluorescent marker has been proven to be a very feasible option. Additionally, labelled PMN can afterwards also be analysed in immunofluorescence experiments in combination with other stainings (e.g. occludin). Another advantage of this experimental setup is that barrier function can be monitored in parallel. In future experiments a similar experimental protocol can be used in the human BCSFB system with cultured HIBCPP.

In conclusion, our inverted cell culture system of the choroid plexus epithelial cells enables investigation of pathogen as well as leukocyte interaction at the BCSFB barrier. The methodology seems applicable and has useful modifications of already-existing systems. Recent functional and morphological analysis of PMN transmigration after bacterial stimulation as well as T-cell transmigration after viral infection support the value of this system. Especially the human model offers now a wide range of options for analysis of CNS disease-related mechanisms at the choroid plexus epithelium.

## Abbreviations

BBB: Blood-brain barrier; BCECF-AM: 2’,7’-bis-(2-carboxyethyl)-5-(and-6)-carboxyfluorescein, acetomethyl ester; BCSFB: Blood-cerebrospinal fluid barrier; CNS: Central nervous system; CSF: Blood-cerebrospinal fluid; DAPI: 4‘-6-diamidino-2-phenylindole dihydrochloride; EAE: Experimental autoimmune encephalomyelitis; HIBCPP: Human choroid plexus papilloma cells; IL-8: Interleukin-8; MCP-1: Monocyte chemotactic protein-1; MS: Multiple sclerosis; MW: Molecular weight; PCPEC: Primary porcine choroid plexus epithelial cells; PMN: Polymorphonuclear neutrophils; TEER: Transepithelial electrical resistance; TJs: Tight junctions; TM: Transmigration.

## Competing interests

The authors declare that they have no competing interests.

## Authors’ contributions

TT conceived and coordinated the study, and drafted the manuscript. CW, US cell culture and immunofluorescence experiments. JB and US performed the electron microscopic studies. CS and HS have co-conceived the study and have been involved in drafting the manuscript. All authors have read and approved the final version of this manuscript.
